# Hydrogen-Bonding Linkers Yield a Large-Pore, Non-Catenated, Metal-Organic Framework with pcu Topology

**DOI:** 10.3390/molecules25030697

**Published:** 2020-02-06

**Authors:** Mohammad S. Yazdanparast, Victor W. Day, Tendai Gadzikwa

**Affiliations:** 1 Department of Chemistry, Kansas State University, Manhattan, KS 66506, USA; yazdanparast@ksu.edu; 2 Department of Chemistry, University of Kansas, Lawrence, KS 66045, USA; vwday@ku.edu

**Keywords:** metal-organic framework, mixed-ligand, pillared, paddle-wheel, non-catenated, large-pore, hydrogen-bonding

## Abstract

Pillared paddle-wheel-based metal-organic framework (MOF) materials are an attractive target as they offer a reliable method for constructing well-defined, multifunctional materials. A drawback of these materials, which has limited their application, is their tendency to form catenated frameworks with little accessible volume. To eliminate this disadvantage, it is necessary to investigate strategies for constructing non-catenated pillared paddle-wheel MOFs. Hydrogen-bonding substituents on linkers have been postulated to prevent catenation in certain frameworks and, in this work, we present a new MOF to further bolster this theory. Using 2,2′-diamino-[1,1′-biphenyl]-4,4′-dicarboxylic acid, BPDC-(NH_2_)_2_, linkers and dipyridyl glycol, DPG, pillars, we assembled a MOF with **pcu** topology. The new material is non-catenated, exhibiting large accessible pores and low density. To the best of our knowledge, this material constitutes the **pcu** framework with the largest pore volume and lowest density. We attribute the lack of catenation to the presence of H-bonding substituents on both linkers.

## 1. Introduction

**Figure 1:**
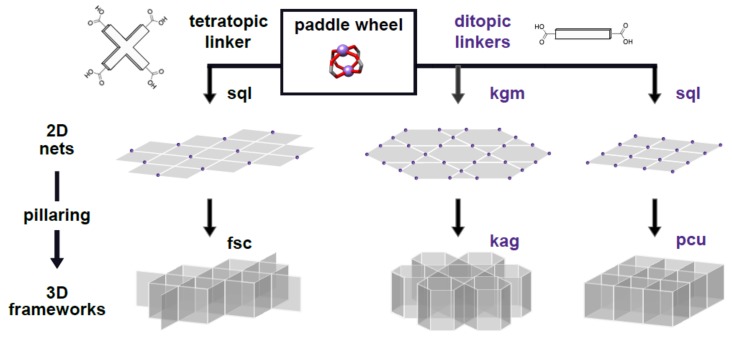
Schematic representation of the possible topologies for pillared, paddle-wheel metal organic frameworks (MOFs). The 2D nets are pillared to form 3D MOFs. MOFs of **pcu** and **fsc** are derived from **sql** nets, and **kag** MOFs are derived from **kgm** nets.

While there are a variety of ways to assemble well-defined, multifunctional metal-organic framework (MOF) materials [1], the construction of mixed-linker, pillared paddle-wheel MOFs is the most efficacious (Figure 1) [2]. Their assembly provides a reliable strategy for introducing two different organic linkers into an MOF, allowing for the chemical pore environment to be tuned with high fidelity [3]. Despite the advantages that they offer, following their first introduction, pillared paddle-wheel frameworks have received much less attention than their potential would warrant. This is owing to two limitations: the M^2+^-paddle-wheel secondary building unit (SBU, Figure 1) is not as chemically stable as many other clusters [4] and, due to the small size of the SBU, the frameworks are prone to catenation. Though the challenges of relatively poor stability and low porosity due to catenation can be addressed post MOF assembly, via transmetallation [5,6,7] and solvent-assisted linker exchange (SALE) [8,9], the de novo synthesis of such materials would be preferable. Thus, there is a need to investigate strategies to incorporate preferred cations and to prevent catenation in the solvothermal synthesis of pillared paddle-wheel MOFs. In this report, we present an unusual, non-catenated, large pore, pillared paddle-wheel MOF, providing an additional datapoint to support current postulation on the factors that may influence catenation in these frameworks.

A major focus of our group is the uniform multifunctionalization of MOFs. To this end, we have been synthesizing pillared paddle-wheel MOFs where the two different linkers bear reactive groups that can be addressed independently post MOF assembly [10,11]. In this work, we specifically target non-catenated frameworks that can accommodate additional functionality. Specifically, we sought pillared frameworks with the **kag** topology as, unlike the more common **pcu**-based structures, they are non-catenated with large pores (Figure 1). For our ligands, we employed dipryridyl glycol, DPG, together with either 2-amino-1,4-benzenediacarboxylic acid (BDC-NH_2_ or 2-azido-1,4-benzenediacarboxylic acid (BDC-N_3_). With the intent of constructing a symmetric version of such MOFs, we then attempted the construction of a **kag** MOF composed of Zn^2+^, 2,2′-diamino-[1,1′-biphenyl]-4,4′-dicarboxylic acid, BPDC-(NH_2_)_2_, and DPG. Gratifyingly, we obtained a non-catenated structure. Unexpectedly, however, we found the structure to have the **pcu** topology.

## 2. Results

**Figure 2:**
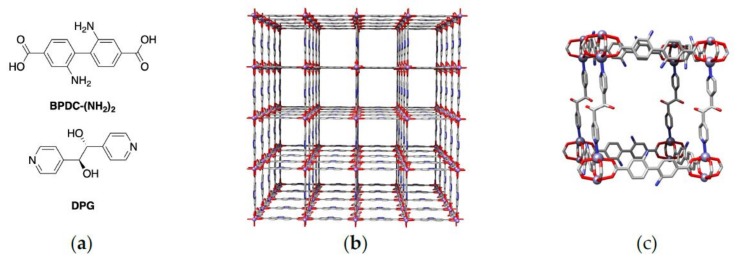
(**a**) MOF linkers; (**b**) **KSU-100** viewed down the c-axis; (**c**) Network unit of **KSU-100**.

Combining BPDC-(NH_2_)_2_, and DPG under the low-temperature nucleation conditions generally employed to obtain **kag** MOFs [12,13], we obtained pale-yellow, block-like crystals that were suitable for single-crystal X-ray analysis. Following refinement of the diffraction data, we found that we had obtained a **pcu** framework, **KSU-100**, that is non-catenated (Figure 2b).

Crystal data for C10H7N1.50O2.50Zn0.50 (M = 220.86 g/mol): monoclinic, space group P4, a = 15.1970(5) Å, b = 15.1970(5) Å, c = 16.2095(5) Å, V = 3743.6(3) Å^3^, Z = 4, T = 200(2) K, μ(CuKα) = 0.542 mm^−1^, D_calc_ = 0.392 g/cm^3^, 33,836 reflections measured (2.908° ≤ 2Θ ≤ 68.403°), 6586 unique (R_int_ = 0.0490) which were used in all calculations. The final R1 was 0.1026 (I > 2σ(I)) and wR2 was 0.2860 (all data).

**Figure 3:**
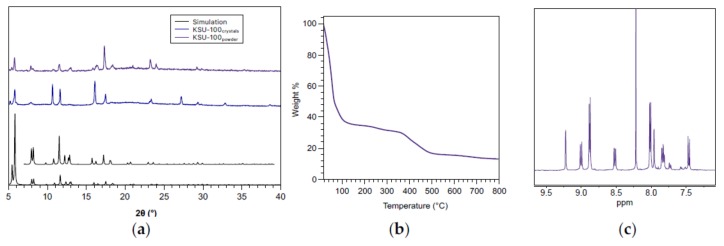
Characterization of **KSU-100**: (**a**) Powder diffraction patterns of the simulated pattern based on the single-crystal data, single crystals, and powder of **KSU-100**. The insert is a magnification of the smaller peaks of the simulation; (**b**) Thermogravimetric analysis (TGA) trace of the MOF solvated with DMF; (**c**) ^1^H-NMR of **KSU-100** digested in a TFA-*d*_1_ and *d*_6_-DMSO mixture.

The new MOF, **KSU-100**, has the BPDC-(NH_2_)_2_ linkers connected by Zn paddle-wheel clusters, defining the *xy*-plane in a **sql** net. This 2D net is then pillared together by the DPG ligand to form a **pcu** framework with large pore dimensions of 11Å × 11Å × 9Å, and a low calculated density of 0.392 g/cm^3^. Powder X-ray diffraction (PXRD) of bulk samples of single-crystals of the material confirmed the purity of the structure (Figure 3a, and Figure S1 in Supporting Information for the indexed pattern). Note that large crystals were used instead of powders, as the powders lost solvent rapidly and did not produce adequate diffraction patterns. Thermogravimetric analysis (TGA) indicates that **KSU-100** loses 60% of its weight as solvent. Such a significant loss confirms that the material has a large solvent-accessible volume and supports that the bulk material is indeed non-catenated (Figure 3b).

To confirm the composition of the material, we performed proton nuclear magnetic resonance (^1^H-NMR) spectroscopy of **KSU-100** digested in a mixture of deuterated trifluoroacetic acid, TFA-*d_1_*, and deuterated dimethylsulfoxide, DMSO-*d*_6_ (Figure 3c). Integration of the ligand peaks (Figure S2) indicates a BPDC-(NH_2_)_2_:DPG ratio of 2:1, as is to be expected for a pillared paddle-wheel MOF. Note that there are two sets of protons corresponding to BPDC-(NH_2_)_2_. Literature precedence indicates that the ligand undergoes multiple transformations in the presence of metal cations and strong acid [14]. We found that using TFA as the digestion acid reduced the number of complexes formed, allowing us to identify and integrate the peaks.

## 3. Discussion

Pillared paddle-wheel MOFs comprise M^2+^-acetate centers connected by multicarboxylate linkers to form two possible two-dimensional (2D) nets: **sql** and **kgm** (Figure 1) [15]. When the paddle-wheel nets are pillared together by ditopic linkers they form 3D frameworks. Of the three pillared paddle-wheel MOF topologies that can be formed, only the **kag** topology, formed from the **kgm** net, has consistently been non-catenated. While low temperature nucleation has been suggested as a method for generating these wide-channel MOFs [13], this topology is relatively rare, with a handful of reports in the last several years [10,11,12,13,16,17]. The more common **sql** net, formed by di- and tetratopic dicarboxylate ligands, is pillared to form the **pcu** and **fsc** nets, respectively [15].

Of the **sql**-based structures, the **fsc** frameworks constructed with tetracarboxy linkers have thus far provided the most reliable route to large-pore, non-catenated, pillared paddle-wheel MOFs. The topology has been primarily reported with the tetrakis(4-carboxyphenyl)porphine (TCPP) [18,19,20,21] and tetrakis(4-carboxyphenyl)benzene, TCPB [22]. The TCPP-based MOFs are all non-catenated, but the TCPB linker produces a net with openings that are large enough to accommodate an additional framework, resulting in frequent catenation [22,23]. Catenation has been prevented by the presence of blocking functional groups on the tetracarboxylate [8], or by using dipyridyl pillars that are bulky or have bulky substituents [24,25,26]. The exception to this trend is **DO-MOF**, a structure where the dipyridyl linker is the seemingly inobtrusive dipryridyl glycol, DPG [27].

The **pcu** topology is generally catenated, unless the MOF is composed of short dicarboxylate linkers or short pillars. The use of short linkers results in frameworks that lack the space to accommodate additional frameworks, thus the lack of catenation comes at the expense of larger pore volumes [28]. The single exception is a non-catenated **pcu** framework, **BMOF-1-bpdc-NO_2_**, composed of bipyridine (BIPY) pillars, and a long 4,4′-biphenyl dicarboxylate (BPDC) linker bearing a nitro substituent [29]. For the TCPB-based MOF, it has been speculated that the hydrogen-bonding capability of the DPG linker is responsible for preventing catenation in **DO-MOF** [8]. That result, combined with the existence of a large-pore, non-catenated **pcn** framework that is decorated with H-bond accepting nitro groups, prompts the question of whether catenation can be influenced by substituents that participate in hydrogen bonding. The new MOF, **KSU-100**, lends credence to this theory by presenting another large-pore, non-catenated **pcu** MOF, constructed using linkers bearing H-bonding substituents.

While we cannot identify the limits of pore dimensions for the construction of **pcu** type MOFs that are non-catenated, it is extraordinary that a **pcu** MOF with a BPDC-based linker is non-catenated. For the small dicarboxylate linker benzene dicarboxylate (BDC), catenated **pcu** MOFs are formed when sufficiently long pillars are used, including the relatively short BIPY [30,31,32,33,34,35]. The same is true for the slightly longer naphthalene dicarboxylate (NDC) linker [36], which still forms catenated structures when the dipyridyl liker has bulky (trimethylsilyl)ethynyl substituents [37]. Even the small, 4-carbon fumarate (FMA) linker forms a catenated **pcu** MOF with the one-ring pyrazine (PYZ) pillar [38]. Given the prevalence of catenation even with short dicarboxylates, it is expected that **pcu** structures of the longer BPDC linker should be catenated.

There is only one report of a non-catenated **pcu** structure of BPDC and a non-hydrogen-bonding pillar, and it is one where the co-linker is the short and bulky DABCO [39]. All other **pcu** structures of BPDC are, at minimum, 2-fold catenated, even when the dipyridyl linker has bulky substituents. Sterically demanding substituents on pillars include anthracene [40] and a 24-member interlocking ring [41], and these pillars have formed 2-fold catenated **pcu** MOFs with BPDC. A pillar containing the bulky triptycene moiety results in a 4-fold catenated MOF with BPDC [35]. Given these examples, and the lack of catenation in **KSU-100** and in **BMOF-1-bpdc-NO_2_**, it is reasonable to assume that it is the electronic nature of the substituents, not their size, that prevents catenation.

Hupp and co-workers [23], and others [42], have suggested that H-bonding between linker substituents and solvent molecules can increase the steric requirements of linkers, preventing catenation. It should be noted that that there is a **pcu** structure that is 3-fold catenated despite having DPG as a pillar [43]. The dicarboxylate ligand in this case is azobenzene-4,4′-dicarboxylic acid, a linker that is only ~2 Å longer than **BPDC**. This result suggests that this may be where the threshold void volume exists, i.e., where the H-bonding capability of DPG is no longer sufficient to prevent catenation. In **KSU-100**, the BPDC-(NH_2_)_2_ linkers and the DPG pillar each have two H-bonding substituents, resulting in a dense concentration of H-bond donors and acceptors in the framework. Such an environment is conducive to the creation of a dense network of H-bonded solvent molecules in the pores. We presume that this is why catenation does not take place despite the significant void volume.

## 4. Materials and Methods

All chemicals were used as received from commercial sources unless otherwise noted. Meso-α,β-di(4-pyridyl) glycol (DPG) was purchased from TCI America (Portland, OR, USA). *N*,*N*-dimethylformamide (DMF) was purchased from Fisher Scientific (Pittsburgh, PA, USA), and zinc nitrate hexahydrate from Strem Chemicals (Newburyport, MA, USA). Dimethyl sulfoxide-d_6_ (*d*_6_-DMSO, 99.9 atom % D) was purchased from Cambridge Isotope Laboratories (Tewksbury, MA, USA), while trifluoroacetic acid-d (TFA-*d1* 99.5 atom % D) was purchased from Sigma-Aldrich (St Louis, MO, USA). 2,2′-Diaminobiphenyl-4,4′-dicarboxylic acid (BPDC-(NH_2_)_2_) was synthesized following a literature procedure [14].

Synthesis of **KSU-100**: in a 500 mL round-bottom flask, Zn(NO_3_)_2_·6H_2_O (400.0 mg, 0143 mmol) and DPG (160.0 mg, 0.74 mmol) were added to 250 mL DMF and stirred at RT for 30 min. BPDC-(NH_2_)_2_ (400.0 mg, 0.147 mmol) was added to the mixture and left to stir at room temperature for 10 min. The flask was then incubated at 60 °C. After 14 h, the flask was removed from the heating block and left at room temperature for 30 h. Pale yellow crystals (300 mg, 30% yield) of the product were collected by filtration and stored in fresh DMF.

Details of single-crystal X-ray analysis are available in the Supporting Information. CCDC 1972127 contains the supplementary crystallographic data for this paper. These data are provided free of charge by the Cambridge Crystallographic Data Centre.

Powder X-ray diffraction (PXRD) patterns were recorded on a Bruker AXS D8 Advance Phaser diffractometer (Bruker AXS GmbH, Karlsruhe, Germany) with Cu Kα radiation (λ = 1.5418 Å) over a range of 5° < 2θ < 40° in 0.02° steps, with a 0.5 s counting time per step. Samples were collected from the bottom of the reaction vial as a thick suspension in DMF and spread on a Si-Einkristalle plate immediately before PXRD measurements.

Thermogravimetric analysis (TGA) was performed on a TGA-Q50 (TA Instruments, New Castle, DE, USA) interfaced with a PC using TA Universal Analysis software. Samples were heated at a rate of 10 °C/min under a nitrogen atmosphere. All samples were extensively solvent-exchanged with fresh DMF prior to analysis.

The proton NMR spectrum of **KSU-100** was recorded on a Bruker Avance NEO spectrometer (400 MHz for ^1^H, Bruker BioSpin, Billerica, MA, USA). NMR chemical shifts are reported in ppm against a residual solvent resonance as the internal standard (δ(*d*_6_-DMSO) = 2.5 ppm). In a typical analysis, MOF materials were washed thoroughly with DMF. The sample was isolated and dried under vacuum at 60 °C for minimum of 2 h. The dry MOF sample (~5 mg) was digested in a mixture of 0.400 mL *d*_6_-DMSO (0.1 mL) and TFA-*d*_1_ (0.100 mL) and then transferred into an NMR tube.

Fourier-transform infrared spectroscopy of **KSU-100** was performed on an Agilent Cary 630 spectrometer (Agilent Technologies, Santa Clara, CA, USA). The MOF sample (~1 mg) was combined with five mass equivalents (~5 mg) of KBr and ground together to a fine powder.

## 5. Conclusions

In our own work of covalently functionalizing MOFs post-synthesis, it has been crucial to synthesize non-catenated frameworks that have enough space to accommodate additional functionality. Doubtless, accessible pore volume is necessary for a variety of other MOF applications. In this work, we have provided an additional datapoint to support the assertion that hydrogen-bonding substituents on linkers can prevent catenation in pillared, paddle-wheel MOFs. With this information, MOF chemists who are interested in the well-defined multifunctionality of these materials now have a potential avenue for constructing non-catenated variants of these pillared frameworks.

## Supplementary Materials

The following are available online, Crystallographic Information File (CIF) for **KSU-100** and Supporting Information.
