# Supplementary material for: Hydrogen-Bonding Linkers Yield a Large-Pore, Non-Catenated, Metal-Organic Framework with pcu Topology
Source: Molecules. 2020 Feb 6;25(3):697. doi: 10.3390/molecules25030697 (PMC7037358; doi:10.3390/molecules25030697)
Supplement: Supplementary file 1 [file molecules-25-00697-s001.pdf]

Hydrogen-Bonding Linkers Yield a Large-Pore, Non-Catenated, Metal-Organic Framework with pcu Topology

Mohammad Yazdanparast[1], Victor W. Day[2] and Tendai Gadzikwa[2,*]

* Correspondence: gadzikwa@ksu.edu

Department of Chemistry, Kansas State University, Manhattan, Kansas 66506, USA.

POWDER X-RAY DIFFRACTION (PXRD) 2

PROTON NUCLEAR MAGNETIC RESONANCE (^{1}H NMR) 2

FOURIER-TRANSFORM INFRARED SPECTROSCOPY (FTIR) 3

SINGLE-CRYSTAL X-RAY CRYSTALLOGRAPHY ANALYSIS FOR KSU-100 (CCDC 1972127) 3

REFERENCES 6

POWDER X-RAY DIFFRACTION (PXRD)

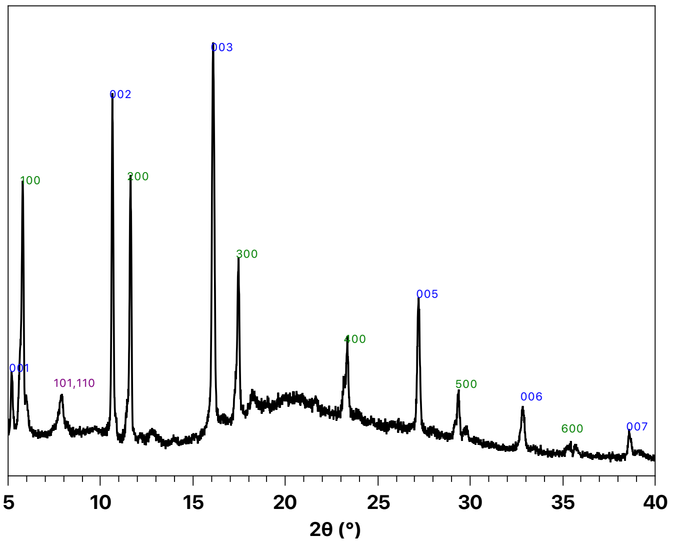

Figure S1. Indexed PXRD pattern for crystals of **KSU-100**.

PROTON NUCLEAR MAGNETIC RESONANCE (^{1}H NMR)

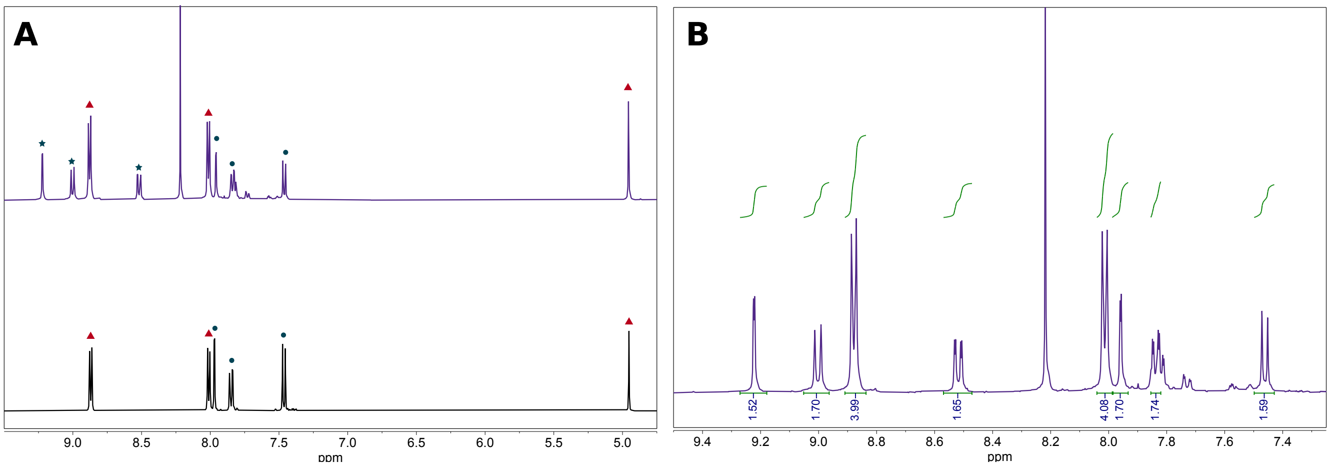

Figure S2. ^{1}H-NMR spectra in TFA/d_6-DMSO. A: Top: **KSU-100**. Bottom: mixture of ligands. DPG (red triangles), BPDC-$(NH_2)_2$ (blue circles), and BPDC-$(NH_2)_2$ following Zn-mediated reaction with acid (blue stars and pentagons).[1] B: **KSU-100** integrated.

FOURIER-TRANSFORM INFRARED SPECTROSCOPY (FTIR)

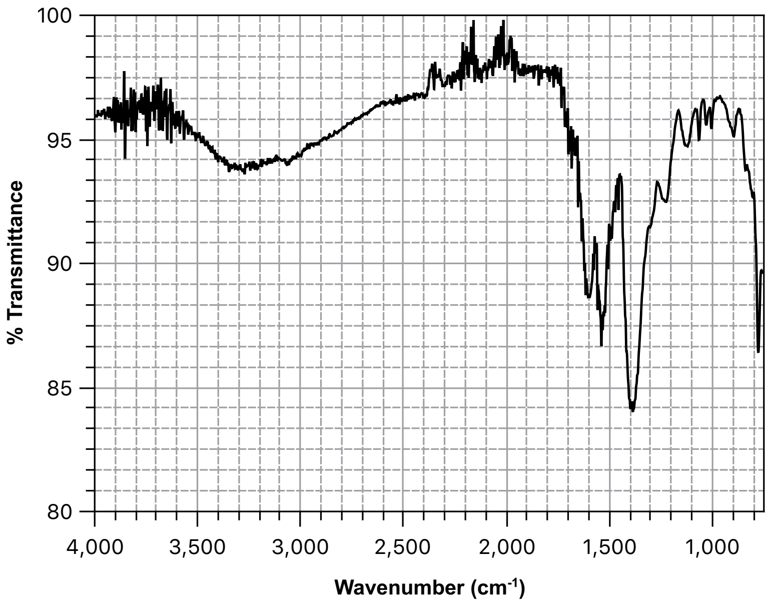

Figure S3. FTIR spectrum for **KSU-100**.

SINGLE-CRYSTAL X-RAY CRYSTALLOGRAPHY ANALYSIS FOR KSU-100 (CCDC 1972127)

A set of diffraction data [33836 reflections using 2410 1°-wide ω- or ϕ-scan frames with scan times of 10-60 seconds] was collected[2] for a single-domain crystal of **KSU-100** using monochromated CuKα radiation (λ= 1.54178 Å) on a Bruker Proteum Single Crystal Diffraction System with dual CCD detectors and associated Helios high-brilliance multilayer optics and a shared Bruker MicroSTAR microfocus Cu rotating anode x-ray source operating at 45kV and 60mA. Data for **KSU-100** was collected with a Platinum 135 CCD detector and a crystal-to-detector distance of 50 mm. The integrated data[3] were corrected empirically for variable absorption effects using equivalent reflections. The Bruker software package SHELXTL was used to solve the structure using "direct methods" techniques. All stages of weighted full-matrix least-squares refinement were conducted using F_o^2 data with the SHELXTL XL v2018 Version 1 software package.[4] Final crystallographic details are summarized in Table S1.

The asymmetric unit of **KSU-100** contains two special-position Zn^{2+} cations [C_4 axis at (½, ½, z)], one four-fold disordered [about the C_4 axis at (½, ½, z)] neutral meso-α,β-di(4-pyridyl) glycol (**DPG**) moiety and half of a fourfold disordered 2,2'-diamino-[1,1'-biphenyl]-4,4'-dicarboxylic acid (**BPDC(NH$_2$)$_2$**),dianion. Although disorder is common and not unexpected in MOF structures, the bridging **DPG** ligand in this particular system has additional potential for inherent disorder. Octahedral coordination at the Zn ions will preclude much rotational disorder of the planar chelating **BPDC(NH$_2$)$_2$** ligand about the Zn••••Zn vector, but each "locked" ligand can still bridge the same two Zn metals in four different ways. Since the **DPG** ligand coordinates to a Zn atom through a single nitrogen on each end, it could have different rotational orientations

about the Zn••••Zn vector. The ligand could also bridge the same two metals with its two ends interchanged. The **BPDC(NH$_2$)$_2$** ligand in **1** is disordered different half-occupancy NH_2 substituents and the **DPG** ligand appears to have fourfold rotational disorder about the Zn••••Zn vector.

Fourier maps from the initial structure solution clearly revealed the Zn atoms, all **BPDC(NH$_2$)$_2$** C and O atoms and the pyridine rings of the **DPG** ligand. Non-structured (presumably disordered) electron density was present between the two pyridine moieties. After refining these clearly identified atoms with isotropic thermal parameters, the Zn and **BPDC(NH$_2$)$_2$** carbon and oxygen atoms were refined with anisotropic thermal parameters. A difference Fourier revealed low electron density peaks at sp^2-hybridized positions for the C24 and C26 carbons of the **BPDC(NH$_2$)$_2$** ligand. These were peaks were included into the structural model as half-occupancy nitrogen atoms N3 and N3'. The difference Fourier also contained a more structured region of electron density between the two coordinated pyridine ligands.

The published structure of meso-α,β-di(4-pyridyl) glycol (**DPG**) was then used with the coordinates of the two pyridine ligands and the disordered electron density between them to derive coordinates for a bridging mode for the **DPG** ligand in **1**. Bond lengths and angles for the atoms of this **DPG** bridging mode were required to have values similar to the published structure and this was then included as a rigid group in subsequent least-squares refinement cycles with variable isotropic thermal parameters for all nonhydrogen atoms except C1 that was fixed at the average value of its covalently bonded nonhydrogen atoms.

Since dual amine sites (N3 and N3') appeared to be present, the **BPDC(NH$_2$)$_2$** nonhydrogen atoms were presumably disordered but still reasonably well-defined. All of the **BPDC(NH$_2$)$_2$** carbon atoms and oxygen atom O1 were therefore included in the structural model with anisotropic thermal parameters. With all of the **DPG** nonhydrogen atoms linking the pyridine rings being disordered and not as well-defined as the **BPDC(NH$_2$)$_2$** atoms, the **DPG** ligand nonhydrogen atoms were included in the structural model with isotropic thermal parameters as were the two disordered half-occupancy amino group nitrogen atoms (N3 and N3') and **BPDC(NH$_2$)$_2$** oxygen atom O2. Hydrogen atoms bonded to carbon or nitrogen in all ligands were included in the structural model as idealized riding model atoms (assuming sp^2- or sp^3-hybridization of the carbon and nitrogen atoms and C-H bond lengths of 0.95 Å or 0.96 Å and N-H bond lengths of 0.88 Å). Hydrogen atoms bonded to hydroxyl oxygen atoms were included as idealized atoms (assuming sp^3-hybridization of the oxygen atom and an O-H bond length of 0.85 Å) with a “staggered” orientation. The isotropic thermal parameters of idealized hydrogen atoms in all ligands were fixed at values 1.2 (bonded to C or N) or 1.5 (bonded to O) times the equivalent isotropic thermal parameter of the carbon, nitrogen or oxygen atom to which they are covalently bonded. Significant residual difference Fourier electron density did not appear at reasonable positions in the MOF channels. Substantial structural voids were therefore present. Refinement of racemic twinning revealed the crystal was a 57/43 racemic twin.

Table 1. Crystal Data and Structure Refinement for $Zn(C_{14}H_{10}N_2O_4)_2(C_{12}H_{12}N_2O_2)$.

Identification code	v58e	
Empirical formula	$C_{10} H_7 N_{1.5} O_{2.5} Zn_{0.5}$	
Formula weight	220.86	
Temperature	200(2) K	
Wavelength	1.54178 Å	
Crystal system	Tetragonal	
Space group	P4-C_4^1 (No. 75)	
Unit cell dimensions	a = 15.1970(5) Å	α = 90°
	b = 15.1970(5) Å	β = 90°
	c = 16.2095(5) Å	γ = 90°
Volume	3743.6(3) $Å^3$	
Z	4	
Density (calculated)	0.392 g/cm^3	
Absorption coefficient	0.542 mm^{-1}	
F(000)	450	
Crystal size	0.370 x 0.220 x 0.130 mm^3	
Theta range for data collection	2.91° to 68.40°	
Index ranges	-18≤h≤17, -15≤k≤18, -19≤l≤19	
Reflections collected	33836	
Independent reflections	6586 [R_{int} = 0.049]	
Completeness to theta = 66.00°	99.7 %	
Absorption correction	Multi-scan	
Max. and min. transmission	1.000 and 0.798	
Refinement method	Full-matrix least-squares on F^2	
Data / restraints / parameters	6586 / 1 / 96	
Goodness-of-fit on F^2	1.005	
Final R indices [I>2σ(I)]	R_1 = 0.103, wR_2 = 0.275	
R indices (all data)	R_1 = 0.118, wR_2 = 0.286	
Absolute structure parameter	0.43(19)	
Extinction coefficient	0.0072(8)	
Largest diff. peak and hole	1.18 and -0.75 $e^-/Å^3$	

REFERENCES

(1) Ko, N.; Hong, J.; Sung, S.; Cordova, K. E.; Park, H. J.; Yang, J. K.; Kim, J. A Significant Enhancement of Water Vapour Uptake at Low Pressure by Amine-Functionalization of UiO-67. *Dalton Trans.* **2015**, *44* (5), 2047–2051. https://doi.org/10.1039/C4DT02582B.

(2) Data Collection: SMART Software in APEX2 V2014.11-0 Suite. Bruker-AXS, 5465 E. Cheryl Parkway, Madison, WI 53711-5373 USA.

(3) Data Reduction: SAINT Software in APEX2 V2014.11-0 Suite. Bruker-AXS, 5465 E. Cheryl Parkway, Madison, WI 53711-5373 USA.

(4) Refinement: SHELXTL Software in APEX2 V2014.11-0 Suite. Bruker-AXS, 5465 E. Cheryl Parkway, Madison, WI 53711-5373 USA.
